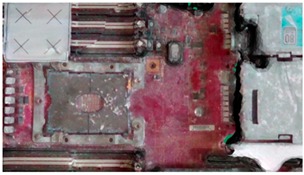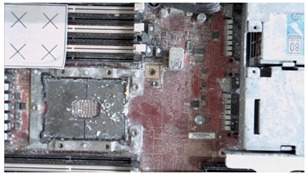# Autonomous Robot-Guided Inspection System Based on Offline Programming and RGB-D Model

**DOI:** 10.3390/s18114008

**Published:** 2018-11-16

**Authors:** Amit Kumar Bedaka, Alaa M. Mahmoud, Shao-Chun Lee, Chyi-Yeu Lin

**Affiliations:** 1 Department of Mechanical Engineering, National Taiwan University of Science and Technology, Taipei 106, Taiwan; akbedaka@gmail.com (A.K.B.); eng.alaa.mohamed442@gmail.com (A.M.M.); bnbscottlee@gmail.com (S.-C.L.); 2 Taiwan Building Technology Center, National Taiwan University of Science and Technology, Taipei 106, Taiwan; 3 Center for Cyber-Physical System Innovation, National Taiwan University of Science and Technology, Taipei 106, Taiwan

**Keywords:** autonomous inspection system, automatic optical inspection (AOI), RGB-D model, offline programming (OLP), manipulator, image rendering, 3D reconstruction

## Abstract

Automatic optical inspection (AOI) is a control process for precisely evaluating the completeness and quality of manufactured products with the help of visual information. Automatic optical inspection systems include cameras, light sources, and objects; AOI requires expert operators and time-consuming setup processes. In this study, a novel autonomous industrial robot-guided inspection system was hypothesized and developed to expedite and ease inspection process development. The developed platform is an intuitive and interactive system that does not require a physical object to test or an industrial robot; this allows nonexpert operators to perform object inspection planning by only using scanned data. The proposed system comprises an offline programming (OLP) platform and three-dimensional/two-dimensional (3D/2D) vision module. A robot program generated from the OLP platform is mapped to an industrial manipulator to scan a 3D point-cloud model of an object by using a laser triangulation sensor. After a reconstructed 3D model is aligned with a computer-aided design model on a common coordinate system, the OLP platform allows users to efficiently fine-tune the required inspection positions on the basis of the rendered images. The arranged inspection positions can be directed to an industrial manipulator on a production line to capture real images by using the corresponding 2D camera/lens setup for AOI tasks. This innovative system can be implemented in smart factories, which are easily manageable from multiple locations. Workers can save scanned data when new inspection positions are included based on cloud data. The present system provides a new direction to cloud-based manufacturing industries and maximizes the flexibility and efficiency of the AOI setup process to increase productivity.

## 1. Introduction

In modern industries, robots are increasingly required to perform complex tasks. Robot integration involves automating the relevant processes to ensure satisfactory quality. Over several years, robotics has been crucial in loading/unloading, assembly, testing, and inspection processes. The inspection of products is crucial in modern manufacturing processes; products must be inspected for quality assurance purposes. Every industry has some standard methods of inspection, which depend on their products or assembly lines. Some of these methods use conventional manpower on an assembly line, whereas others use state-of-the-art techniques. These methods have provided opportunities for researchers to generate innovative concepts for improving the service quality of inspection, particularly robot-guided automatic optical inspection (AOI).

Although numerous studies have been successfully conducted to perform AOI, no studies have been performed to assess an OLP platform integrated with a 2D/3D vision module to perform autonomous robot-guided optical inspection using an RGB-D model for precisely evaluating the manufactured products. Furthermore, Table 1 presents the performance of this system with the existing AOI system [1,2]. The proposed system expedites and eases the development of automatic optical inspection. Automatic optical inspection engineers can strategize AOI positions and the corresponding robot trajectory regardless of time and location without the presence of the physical product, industrial manipulator, inspection camera, or lens by only using scanned data. The image must be captured by the camera at an accurate position such that it comprises high-quality content for the working of optical inspection software. A tedious trial-and-error process of changing camera position and observing the captured images is essential to obtain appropriate image content. This process requires considerable time for using a teach pendant to vary the position of the camera. This situation is worsened when an AOI engineer, who must determine the final camera position for each inspection location, is asked to perform a robot teaching process. In the proposed system, the robot position can be conveniently determined using a virtual robot and product in OPEN CASCADE (OCC) software. This method reduces the complexity of system development for managing the movement of a robot carrying a camera and lens. In addition, this system can be easily managed from multiple locations and allows users to define new optical inspection positions from cloud data based on scanned information without the presence of the physical object. Whereas, exiting AOI systems still requires physical set-up to perform inspections, which makes it more challenging to manage from multi-locations.

### 1.1. Offline Programming

In previous years, numerous studies have explored efficient applications of industrial robots with intelligent modules to replace traditional manual manufacturing operations. However, using a traditional teach pendant method to generate a required robot trajectory in complicated working situations is tedious. In this traditional method, a tool path is manually generated by an operator. After all points have been recorded, the robot can play back the points to obtain a defined trajectory. To overcome these challenges, offline programming (OLP) software can automatically generate a robot path by using computer-aided design (CAD) models.

An offline programming method generates a 3D virtual environment, wherein a work environment is defined with respect to a real-time scenario. The robot path is automatically generated and graphically simulated to evaluate performance before the software maps the path to an industrial manipulator. The typical OLP method is rapid, cost-effective, time-efficient, and prevents losses by allowing testing in a virtual environment [3,4,5].

Complications related to robot trajectory generation have received considerable attention and provided opportunities for researchers to generate innovative solutions. The introduction of a CAD-based solution has engendered a shift in robotics [6]. Recently, CAD has been intensively used in robotic systems to strategize trajectories and simulate robot performance [7]. A CAD-based graphical simulation platform can provide visualizations of robot motion in virtual environments for simulation of desired tasks [8]. The simulation assists in resolving problems related to collision [9] and singularities [10].

Several commercial software packages, such as KUKA.Sim, RobotStudio, and ROBOGUIDE are now available. The use of these software packages is unfortunately limited to particular robots. Other packages, such as RoboSim, Delmia, RobotDK, and RobCAD can generate robot programs for robots from different manufacturers [8,11]. These software packages automatically generate a robot program and simulate a specific task; however, a license is required to access them. Furthermore, integrating a self-developed program into these commercial packages to work on numerous tasks is essential. Therefore, in this study, an OLP platform was developed based on OCC open source libraries [12]. This platform allows easy integration with other systems to generate novel systems. In addition, it extracts CAD features (e.g., points, curves, and surfaces) to generate a robot path and assists in designing and developing an application-oriented platform to offer an intuitive and interactive environment.

### 1.2. Three-Dimensional Reconstruction Methods

Three-dimensional (3D) reconstruction is crucial in different research areas, such as robotic navigation, animation, reverse engineering, inspection, graphics, object recognition, and computer vision. Three-dimensional reconstruction is the process used for retrieving the 3D profile of a real-time object or scene. Moreover, the complete process from data acquisition to 3D virtual model generation is considered 3D reconstruction [13]. Because it is a fundamental stage, the quality of the 3D reconstructed model must be critically realized.

**Figure 1:**
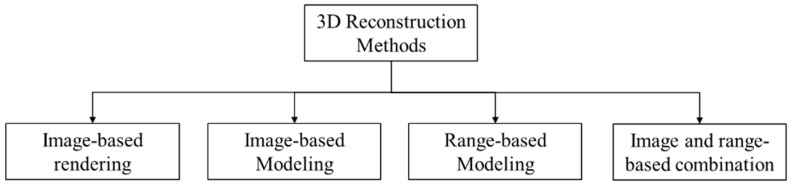
Three-dimensional (3D) reconstructing methods.

Three-dimensional reconstruction can be achieved through different methods using different tools. Such methods include image-based rendering, image-based modeling, range-based modeling, and combinations of image- and range-based methods [14,15,16,17] (Figure 1).

### 1.3. 3D Reconstruction Sensors

**Figure 2:**
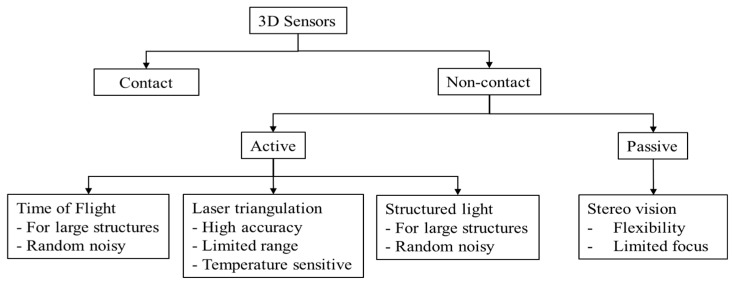
3D sensor categorization technologies, advantages, and disadvantages.

To implement any of the aforementioned methods, a vision-based sensor must be used. These sensors are categorized as contact and noncontact sensors. Contact sensors detect the object through physical contact, whereas noncontact sensors detect objects without physical contact. Nowadays, noncontact sensors are prevalently used for their portability. Noncontact sensors apply various technologies and are categorized as active and passive sensors on the basis of their technologies. Figure 2 presents different 3D sensors with their technologies, advantages, and disadvantages [18,19,20,21,22].

**Figure 3:**
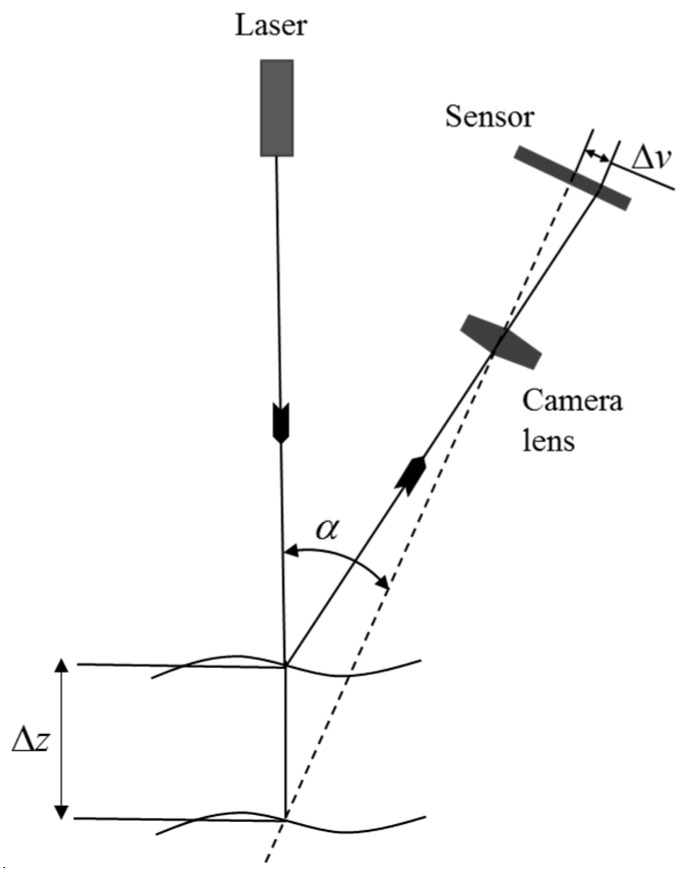
Laser triangulation technology [29]. Source: SCImago, (n.d.). SJR—SCImago Journal & Country Rank [Portal]. Retrieved 5 October 2018, from http://www.scimagojr.com.

Laser scanners accurately obtain large amounts of data in a short time. A laser scanner comprises a laser emitter, receiver, and 2D camera. The laser emitter radiates laser lines or spots on an object, which reflects these radiations to the receiver (Figure 3). The distance between the laser emitter and receiver, and the angle of reflecting laser beams is known. From these known values and by using laser triangulation theory, the distance between the laser scanner and the object can be easily calculated. A 2D camera is an option in some laser scanners and can be used to capture color information [23,24].

### 1.4. Industry 4.0

Industry 4.0 represents the fourth industrial revolution, which defines the current trend of automation and data exchange in manufacturing industries [25]. Industry 4.0 is aimed at creating intelligent factories that are upgraded and transformed using the Internet of Things, cloud computing, and cyber-physical systems (CPS) [26,27]. Industry 4.0 contributes to the triple bottom line in different areas, including order management, research and development, manufacturing processes, and recycling of products for satisfying customer requirements. To address a dynamic and global market, advanced information and manufacturing technologies imbue manufacturing industries with intelligence [28].

**Figure 4:**
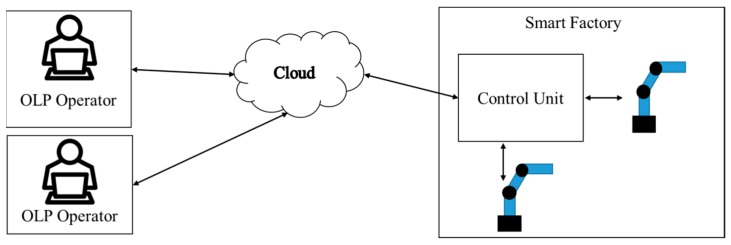
Overview of cloud-based autonomous robot-guided inspection system.

Industry 4.0 includes the integration of technologies for improving the efficiency and receptiveness of a production system at reasonable cost. Therefore, in AOI, an autonomous robot-guided inspection system is developed to reduce human effort and time consumption in an inspection setup process. The cloud system can be easily accessed from multiple locations and allows users to define new inspection positions at any time, without the presence of a physical object (Figure 4). This is a new contribution of Industry 4.0.

In this study, a novel autonomous robot-guided optical inspection system based on the OLP platform was developed by integrating a 2D/3D vision module. The OLP platform was designed and developed using OCC open source libraries with Microsoft Visual Studio. The OLP platform utilizes CAD information to design a 3D scanning trajectory and generate a robot program. Furthermore, simulation results are mapped to an industrial manipulator to reconstruct the 3D model of the object by using the laser triangulation sensor. The developed platform is an intuitive and interactive system that does not require a physical object or an industrial manipulator; this system enables nonexpert operators to perform object inspection by using only scanned data. The reconstructed 3D model is aligned with the CAD model by calculating transformation between inspection positions on a common coordinate system. The OLP platform allows users to define the required inspection positions in a virtual environment. To capture real-time images, position information is provided to the industrial manipulator with the 2D cameras and lenses. The effectiveness and robustness of the proposed system is indicated by comparing sets of real-time captured inspection images and rendering images. In addition, users can virtually select different cameras and lenses to perform inspections without changing the physical setup. This innovative system minimizes human effort and time consumed to expedite the AOI setup process for increasing productivity.

## 2. System Overview

**Figure 5:**
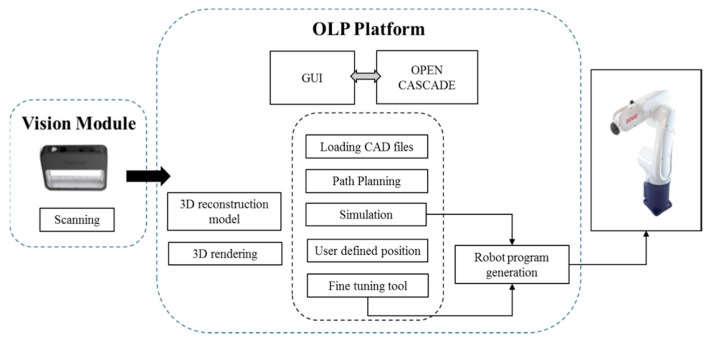
Architecture of proposed autonomous inspection system.

**Figure 6:**
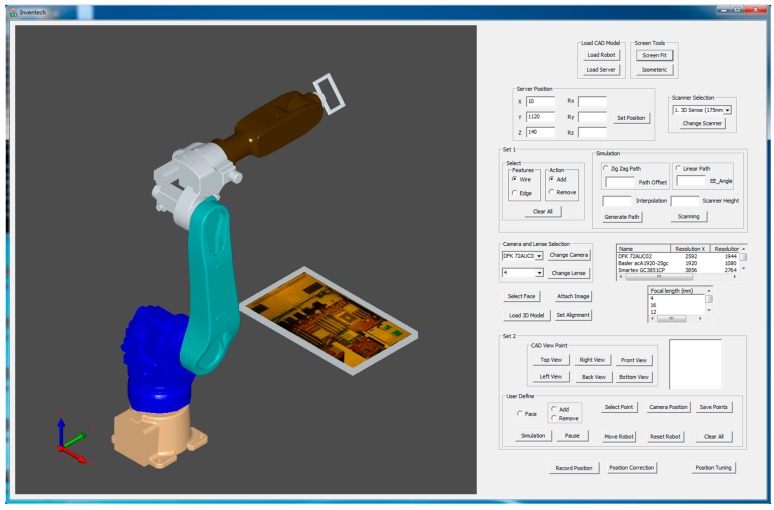
Overview of the proposed software graphic user interface (GUI).

**Figure 7:**
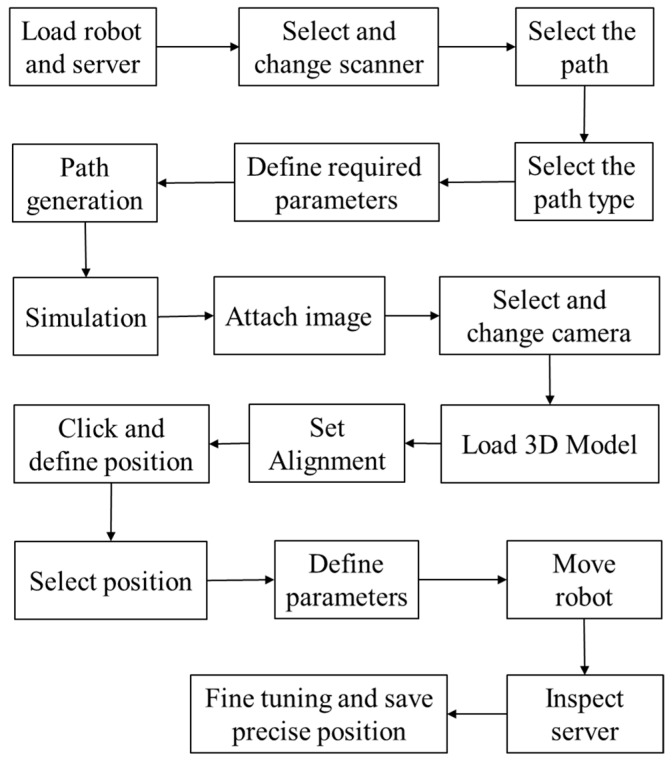
Key steps of (offline programming) OLP platform implementation.

Although the proposed system is particularly applied for inspecting the assembled and misplaced components of a manufactured server for AOI-defined positions [30], this system can be used in other robot-guided AOI applications. Figure 5 presents the overview of the proposed system architecture. On the basis of an approach proposed by Amit et al. [10,31], an application-oriented OLP platform was designed and developed using OCC open source libraries with Microsoft Visual Studio. The OLP platform uses CAD information to generate a path for a robot to perform a scanning task. The platform helps users to evaluate and visualize the generated path through simulation in the virtual environment before mapping to an industrial manipulator. The primary objective of scanning is to reconstruct the 3D model of the server and render 2D inspection images by using the laser scanner. In addition, the platform can create an inspection vision determined by combining embedded scanners, cameras, and lenses on a robot end-effector. Figure 6 presents the design and development of a graphic user interface (GUI). In addition, a server image is attached to the CAD model for user reference (Figure 6). The attached image assists users in evaluating and selecting the required inspection positions and renders 2D images from the reconstructed model corresponding to these positions in the OLP platform. The OLP platform provides robot tuning tools to ensure position precision during inspection. The manipulator then moves to the selected positions and captures real-time images. The presence of two sets of real-time and virtual inspection images captured from the same position assists in evaluating system performance. The flow chart (Figure 7) briefly presents steps to implement the developed system.

## 3. Overview of Proposed OLP Platform

In this study, an application-oriented OLP platform was designed and developed to provide an intuitive and interactive environment by using OCC libraries and CAD kernels. The developed OLP platform allows nonexpert robot operators to perform object optical inspections and manage trajectories by using the scanned data without the physical presence of a server and an industrial manipulator. Figure 5 presents the architecture of the proposed OLP platform. The figure includes crucial steps, such as path planning, user-defined initial position for inspection, and fine-tuning tool for precise positioning, as highlighted in the following sections. In addition, the developed OLP platform is capable of detecting possible collision for industrial robot before mapping to a real site. To evaluate the robustness and effectiveness of the developed OLP platform, paths for server scanning and user-defined AOI positions for server inspection were obtained. Furthermore, simulation results were mapped to the industrial robot for evaluating the performance of the developed system.

### 3.1. Path Planning for Scanning

**Figure 8:**
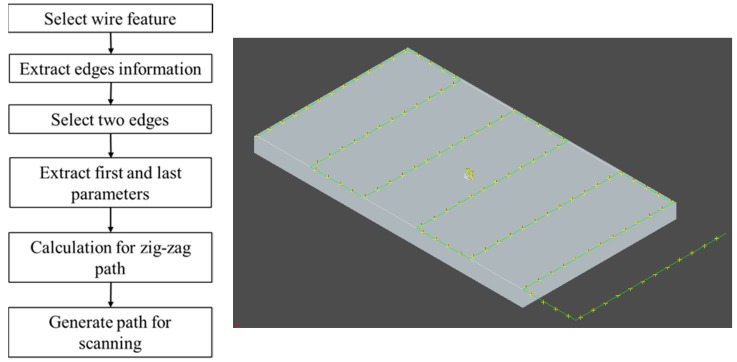
Steps to generate path for server scanning and proposed path.

Path planning starts with the extraction of information from a CAD model. To generate and extract a robot path, understanding the type of information required is essential. In the present approach, OCC libraries are used to extract CAD features for path planning and path generation. The steps and proposed paths in Figure 8 illustrate the path planning and path generation process during scanning. The proposed system utilizes CAD features to plan and generate a path. In this process, a wire feature was detected and selected using the OCC classes TopoDS_Shape and TopoDS_Wire, respectively. Furthermore, edge information was extracted from the wires by using TopExp_Explore. The first and last parameters from the edges were then obtained to perform calculations for path planning before generating a scanning path.

### 3.2. User-Defined Initial Positions for Inspection

**Figure 9:**
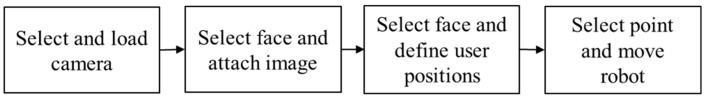
Steps for user-defined positions in server inspection.

**Figure 10:**
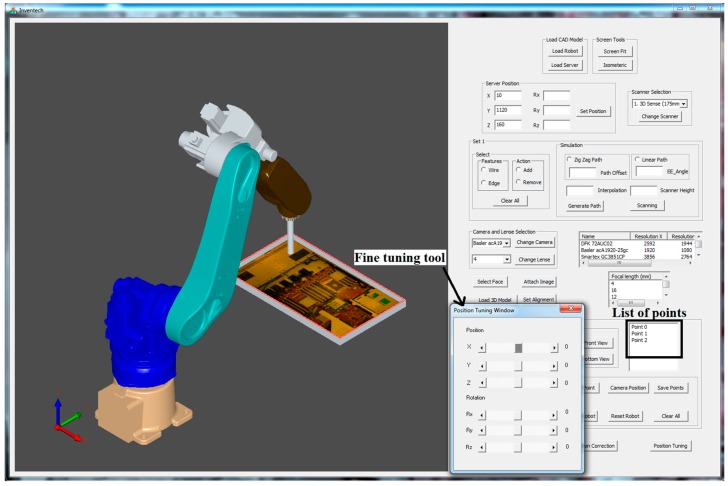
Computer-aided design (CAD) model with sever image and fine-tuning tool.

In the developed system, prior to selecting user-defined initial positions for inspection, the user evaluates camera specifications, uploads them, and follows the steps as shown in Figure 9. The determination of the accurate component position is difficult for a user on a plain CAD model. Therefore, a server picture (Figure 10) is attached on a CAD model surface for reference. On the CAD model with a server photo, the user selects a point on the face upon which the inspection needs to be performed. Furthermore, positions are defined on the server model by clicking the mouse, and the defined positions were generated by utilizing OCC libraries for reference to move a robot in the virtual environment. To move the robot, a point must be selected from a list of points for performing inspection (Figure 10).

### 3.3. Fine Tuning Tool for Precise Position

An effective optical inspection requires an accurately captured image containing all necessary information for inspection. This position must be obtained by trying different camera positions. In the proposed system, the user-defined initial position is not as precise as an inspection position. The user-defined position acts as an initial trial position, and AOI software engineers move the virtual camera from this position to the accurate position. To assist in achieving this goal, fine-tuning tools are provided to translate and rotate a robot with respect to the *x*-axis, *y*-axis, and *z*-axis by using the provided scroll bars in the proposed GUI (Figure 10). When the precise position is obtained, the new inspection position is saved, and this step is repeated for all points from the inspection list.

## 4. Overview of Proposed Vision Module

The present study aims to autonomously inspect the assembly and misplaced components of the manufactured server. The primary objective is to reconstruct a 3D model of the server (target object) by using a laser scanner. Then, align the reconstructed 3D model with the CAD model of the server to render 2D inspection images. These 2D images are rendered based on selected 2D camera types, positions, and focal lengths of lenses. The following sections provide details on 3D scanning and 3D rendering techniques.

### 4.1. 3D Scanning

**Figure 11:**
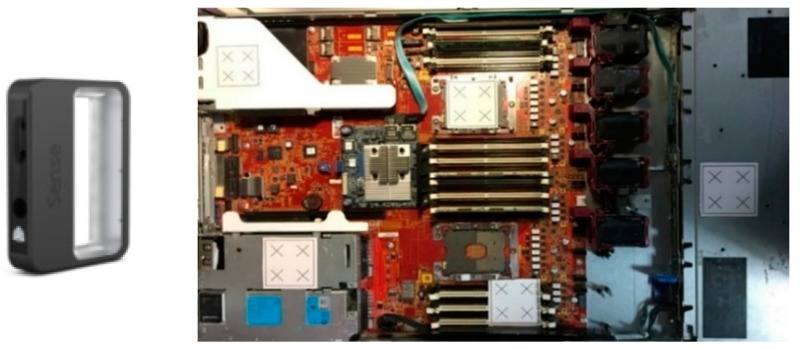
3D sensor and target object industrial server.

**Figure 12:**
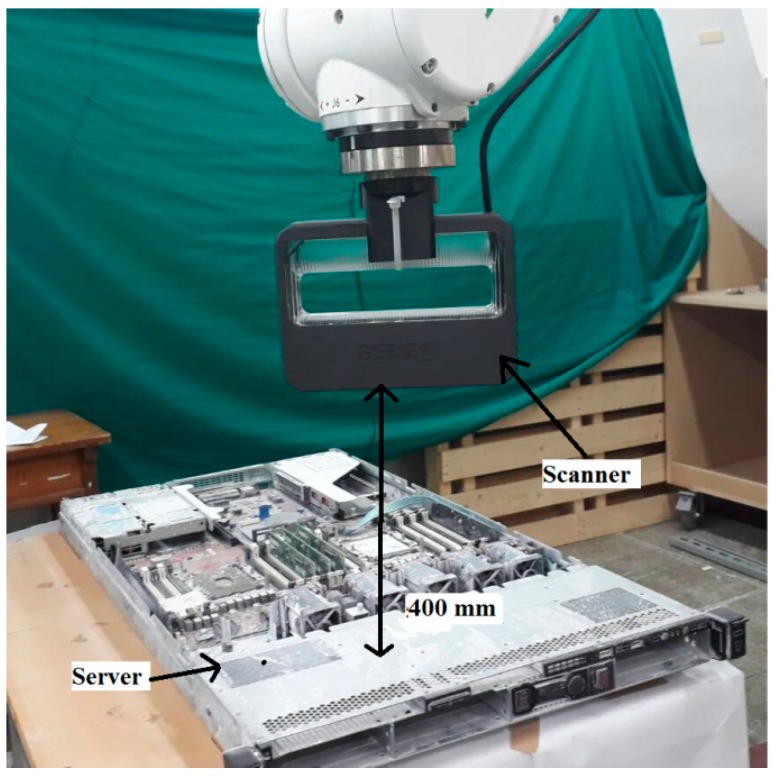
Optimal 3D scanning trajectory requirements.

For all 3D reconstruction methods, the range-based modeling method is performed to reconstruct the 3D model of a real-time server. This method directly captures the 3D geometric and color information of the object by using a 3D sensor. The laser scanner can accurately obtain a large amount of data in a short time, and thus, it is selected as a scanning tool for such industrial applications. After several episodes of trial and error, the initial scanning path is obtained. On the basis of the studies conducted to define optimal scanning conditions, the accuracy of the 3D model depends on the scanning tool and object. The “3D sense” laser scanner is used to scan a 76 × 43 × 4 cm^3^ server (Figure 11). The server comprises a metal base, fans, electronics slots, and screws of different sizes and colors. Although the 3D laser scanner has accuracy, it has some limitations. These limitations are unresolved in the regions containing shiny metal components, or narrow black components. Although the server is feature-rich, it has some metal parts that add more reflection beams than can be expected by the sensor. Moreover, the server contains black fans that absorb laser beams and reduce their reflection to the sensor. The challenge was to use a laser scanner for precisely reconstructing the 3D model for the server. When the scanning trajectory is prudently strategized with the constraints, such as laser offset from the server and laser position and orientation (Figure 12), hybrid techniques [32], including adding laser spots, extra light, and antiglare spray, are used to obtain an accurate 3D model. Laser spots or landmarks add artificial features to the plain areas, and the excessive light illuminates the dark areas, which cannot be evidently observed using the laser scanner. In addition, an antiglare spray moderates the condition by adding nonreflective features, reduces metal reflections, and whitens the dark areas.

### 4.2. Alignment of Reconstructed 3D Model and CAD Model of the Server

**Figure 13:**
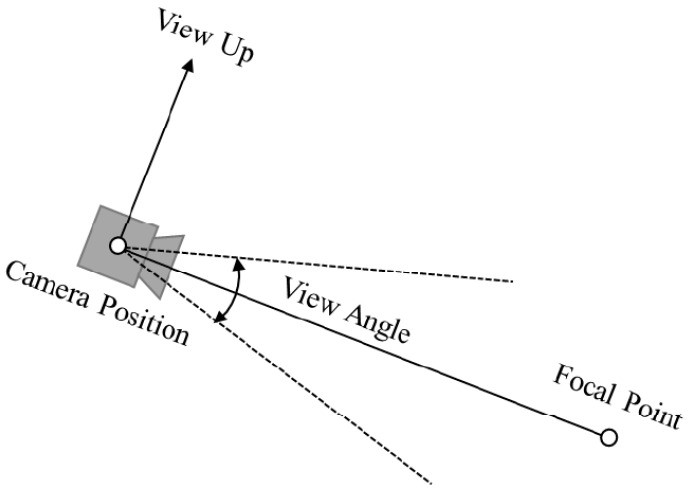
Parameters used to setup the virtual camera in the rendering system.

Before rendering 2D images, aligning the 3D reconstructed model with the CAD model of the server is essential. This alignment assists in rendering the image in the CAD environment at the user-defined position. To setup the virtual camera in the rendering system, three parameters must be defined: camera position, focal point, view up, and view angle (Figure 13). The user-defined position is the target camera position. The focal point and view up are predefined in the system as (0, 0, 100) and (0, −100, 0), respectively.

**Figure 14:**
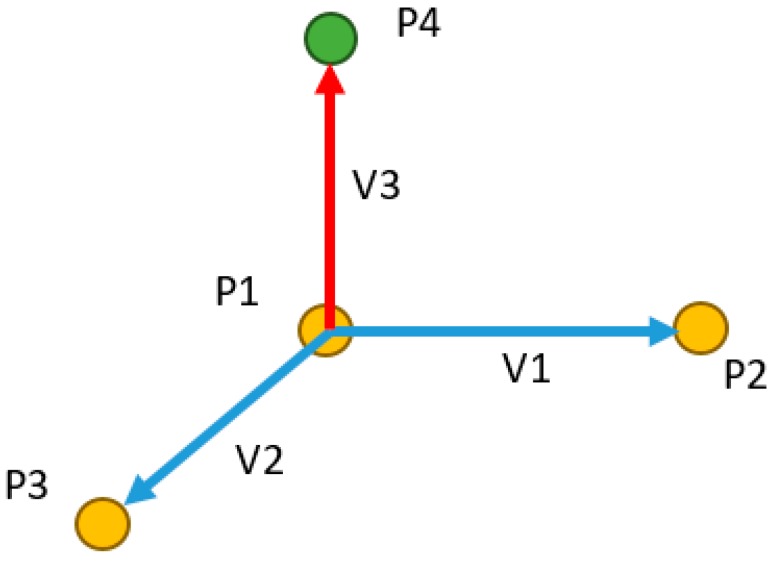
Representation of three points to generate fourth point.

To convert a point from a CAD coordinate system to a camera coordinate system (a rendering coordinate system), a transformation matrix between the two systems is required. In the proposed system, point coordinates are measured in both coordinate systems by using three points from the artificial landmark (Figure 11). Generally, four points are required to calculate the transformation matrix between two coordinate systems. However, in this application, three points (represented in yellow) are used to generate the fourth point (represented in green) (Figure 14), which reduces processing time required to measure the points.

The fourth point is generated using the following Equations (1)–(4):

V1 = P2 − P1,(1)

V2 = P3 − P1,(2)

V3 = cross(V2, V1),(3)

P4 = P1 + V3,(4)

Now, the transformation matrix *T* is computed using the four points:

(5) $$P_{SetB}=\left[T\right]P_{SetA},$$

(6) $$\left[T\right]=P_{SetB}{}^{-1}P_{SetA},$$

where $P_{SetB}$ and $P_{SetA}$ are the points set from two coordinate systems.

Finally, the transformation matrix *T* is obtained using Equations (5) and (6). The camera position, focal point, and view up can now be transformed from the CAD coordinate system to the camera coordinate system by using Equation (7).

(7) $$P_{B}=\left[T\right]P_{A},$$

where $P_{B}$ and $P_{A}$ are the camera parameters in two coordinate systems. On the basis of these parameters, the rendering system can generate the image.

### 4.3. 3D Rendering

**Figure 15:**
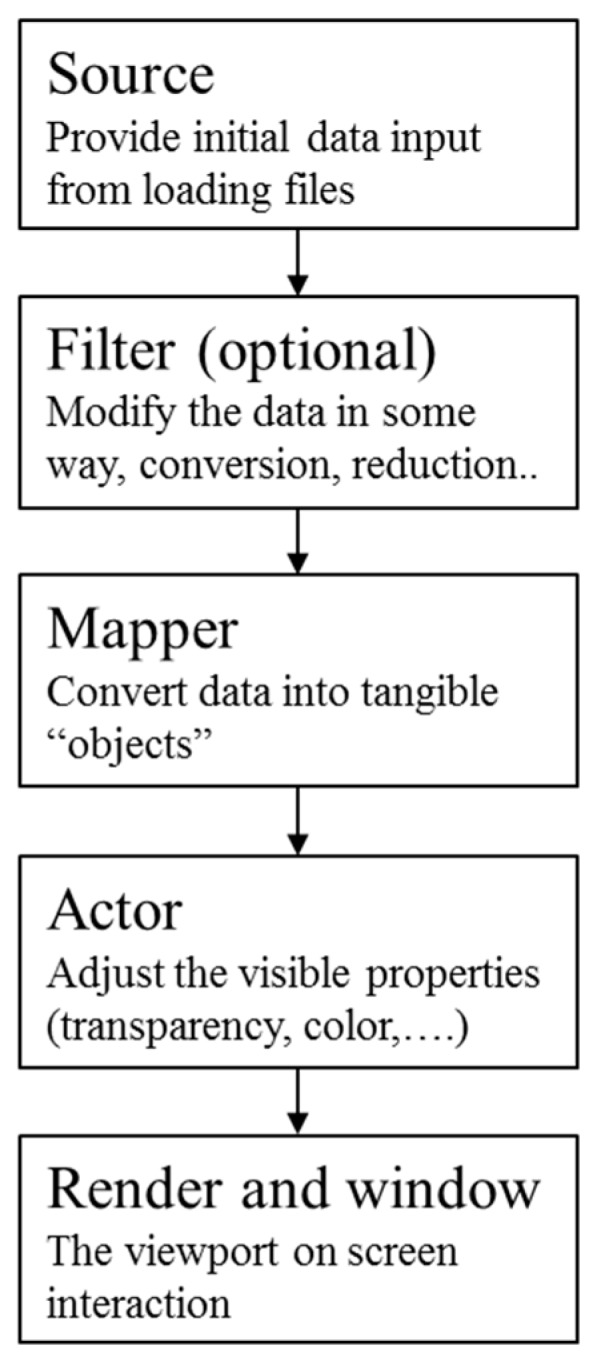
The Visualization Toolkit (VTK) library’s 3D rendering pipeline First item.

After aligning the CAD model and 3D reconstructed model, a 2D image is rendered, and the AOI software engineer then evaluates the accuracy of the content for the particular optical inspection task. The Visualization Toolkit (VTK) is an open source library prevalently used for such graphics application [33,34,35]. In this study, the 3D rendering technique is implemented using the VTK, which follows a structured pipeline to perform the 3D rendering process (Figure 15). The 3D reconstructed model is first loaded as the data source. It is then converted into a “mapper”, which is an object of the VTK, to be used as a graphics primitive. The mapper loads an “actor” for accessing its geometrical and display properties. Illumination is one of the most crucial display parameters because it can significantly change the 3D rendering accuracy. In the present application, white light is adjusted to follow the position and rotation of the virtual camera. Each 2D image is rendered based on the type, position, and the focal length of the lens of the selected virtual camera. From the sensor size and the focal length of lens of the inspection camera, the angle of view is calculated using the following equation:

(8) $$\mathrm{View of angle}\left(\mathrm{degrees}\right)=2\times \tan^{-1}\frac{\mathrm{sensor}\mathrm{width}\left(\mathrm{or}\mathrm{hieght}\right)\mathrm{in}\mathrm{mm}}{2\times \mathrm{focal}\mathrm{length}\mathrm{in}\mathrm{mm}}$$

**Figure 16:**
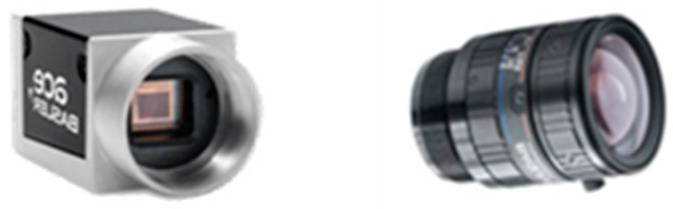
The acA1920-25gc (Basler industry camera) and the C125-0418-5M lens.

A set of industrial camera and lens was used to perform 3D real-time rendering from the virtual systems (Figure 16). acA1920-25gc Basler is the camera’s model with 1920 × 1080 resolution and 0.0022 × 0.0022 mm pixel size. C125-0418-5M, F1.8, f4mm Basler is the lens’s model with 100 mm minimum distance and 5-megapixel resolution. From the previous information and using Equation (8), the view angle was calculated using these data. To evaluate the accuracy of the rendering system, the virtual cameras were set with the same parameters as those of the real cameras. After rendering 2D images, the real camera was moved to the same positions to capture real-time images for comparison.

## 5. Integration of the Rendering Module with the OLP Platform

**Figure 17:**
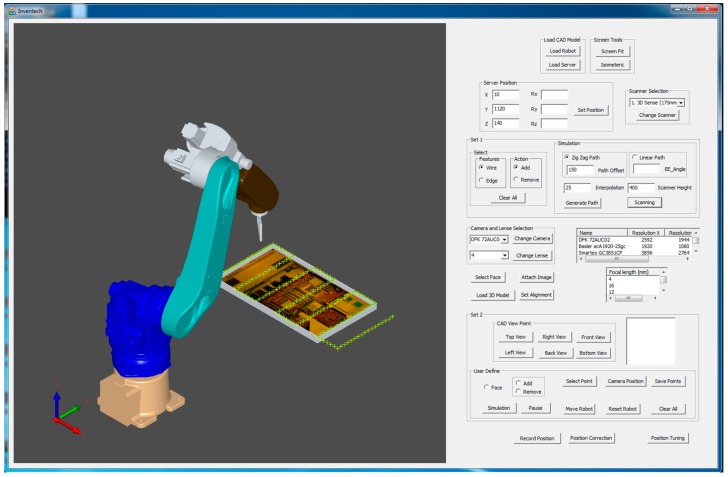
Proposed robot scanning path and simulation.

The developed vision module was integrated with the OLP platform to develop an autonomous robot-guided optical inspection system. Before performing a real-time scanning, path planning was performed to generate a robot path (Figure 17). Furthermore, robot simulation was graphically visualized to generate a robot program and was sent to the HIWIN-620 industrial robot. In this section, the results of the 3D scanning, 3D rendering, and autonomous inspection system are discussed.

### 5.1. OLP Platform Simulation and 3D Scanning Results

In the developed OLP platform, a robot program was generated using CAD information to implement 3D scanning. The required parameters were defined according to the scanning trajectory for all preprocessing items explained in Section 4.1. Finally, the robot path was generated (Figure 17) and simulated in the virtual environment before mapping to the HIWIN robot. Because the 3D scanning result is object-dependent, the details of the target object must be known. The target object was a server comprising metal (reflective) areas, black (absorbent) areas, and small electronic components, and screws (rich-features area). To maximize the accuracy of the 3D reconstructing model, hybrid techniques were implemented, such as adding artificial laser landmarks, increasing light intensity, and spreading an antiglare spray on the object before the scanning process.

**Figure 18:**
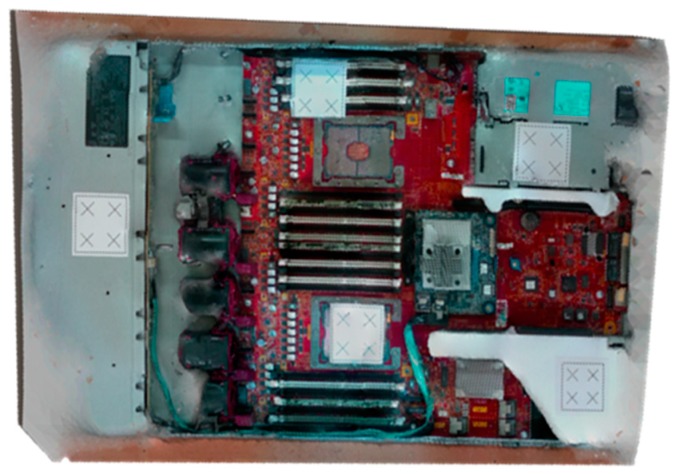
Final 3D reconstructed model for server.

The addition of the excessive light spots helped the sensor to recognize some of the missing parts, particularly in corners. However, increasing the light toward the metal parts increased the glare and shine, which resulted in a noisy model. By contrast, adding colored laser landmarks helped the sensor to stably observe some metal areas because of its color and its ability to reduce the unexpected metal reflections. The spreading of white-colored antiglare spray on the server resulted in the substantial improvement of accuracy (Figure 18). The spreading significantly reduced the reflections in the metal areas and added color to the black fans, which reduced the beam absorption of the laser sensors. However, the borders of servers were difficult to observe. To overcome this limitation, light spots and laser landmarks were used with the antiglare spray. Another critical point that should be considered is the occlusion in laser triangulation method [32]. To overcome it, we carefully fixed the orientation of the sensor initially and during the scanning process to minimize the occluded parts of the object. Also, the 3D sensor’s SDK attached a 2D image on the 3D reconstructed model after finishing the scanning to process. Furthermore, the alignment process was performed with the reconstructed 3D model as explained in Section 4.2.

### 5.2. AOI-Defined Positions and 3D Rendering Results

**Figure 19:**
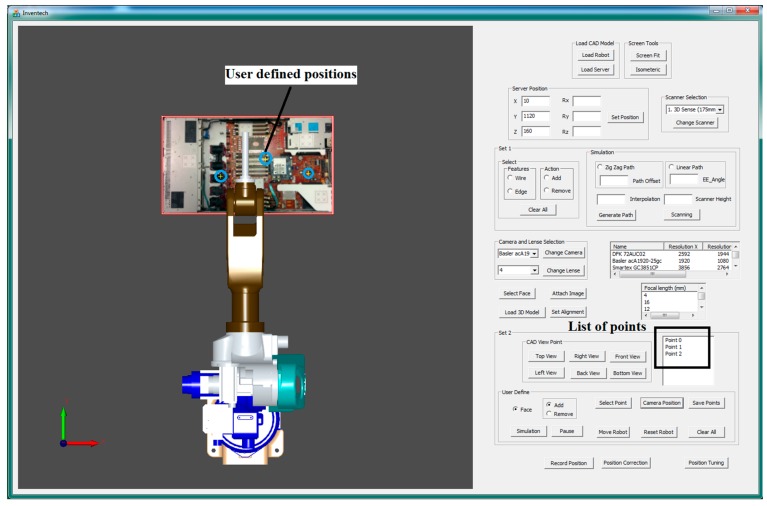
User-defined positions to perform inspection.

**Figure 20:**
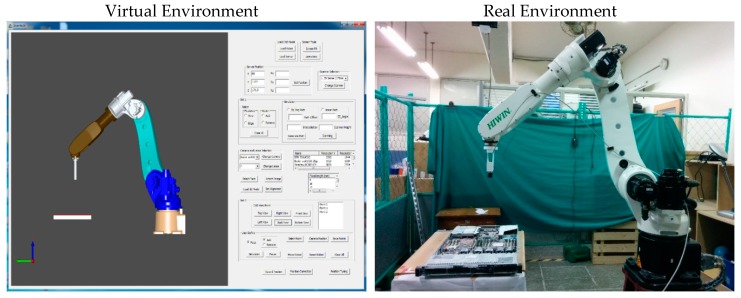
Real-time environment versus virtual environment.

When the reconstructed 3D model and CAD model of the server were aligned, the VTK library was used to render virtual images from it. Virtual images were rendered from the preselected positions of the virtual camera. These positions were selected through a user interface (Figure 19). Then, the acA1920-25gc (Basler GigE industrial cameras) and the 4-mm focal lens were attached to the end-effector of a HIWIN robot to capture real-time images at the user-defined positions. Figure 20 presents the virtual and real-time environments. The parameters of the virtual camera, including the size of the sensor and pixel, resolution, and field of view, were adjusted to match with those of the real-time camera. Table 2 presents the results of the virtual and real-time images captured by the acA1920-25gc and the 4-mm focal lens.

The obtained real-time images significantly matched the virtual images, particularly for the frame ratio and field of view. Because of the differences between the real and virtual environment, it was not possible to adjust the position of the server and the gripper position and rotation of the 2D camera precisely. Therefore, a minor rotation shift and scaling difference of less than 3° and 3 mm depth, respectively, were observed. This shift could be easily resolved by precisely installing the server and camera gripper. In the developed system, by replacing the setup of the real-time camera with that of the virtual camera and replacing the real-time server with the 3D reconstruction model, the time and complexity of the complete inspection trajectory planning process were significantly reduced. This virtual system can be set up in any location without the physical presence of the server and robot, which would be expected to increase productivity and flexibility. The trajectory algorithm in the proposed OLP platform is suitable to scan a planner object (server). On the other hand, scanning a complex geometry requires a sophisticated algorithm to develop the trajectory, which is not supported by the proposed platform. Despite this, the system can perform inspection if the scanned model of a complex object (engine block, containers) is available.

## 6. Conclusions

In this study, a novel autonomous robot-guided optical inspection system based on an OLP platform was developed by integrating the 2D/3D vision module. The OLP platform was designed and developed using OCC open source libraries with Microsoft Visual Studio. The proposed OLP platform utilized CAD information to strategize a 3D scanning trajectory and generate a robot program. Furthermore, simulation results were mapped to an industrial manipulator to reconstruct the 3D model of the object by using a laser triangulation sensor. To obtain the accurate 3D reconstructed model of the server, preprocessing was performed before scanning. The VTK library was used to render the virtual images from the scanned model through the virtual camera for the user-defined targets after aligning the 3D reconstructed model with the CAD model. When the positions were defined on the server, real-time images corresponding to the positions were captured using the industrial manipulator. In addition, users can perform inspections by selecting different cameras and lenses virtually, without changing the physical setup. To evaluate the effectiveness and robustness of the proposed inspection system, a set of virtual inspection images were compared with the set of real-time images. The proposed system had minor rotation shift and scaling difference of less than 3° and 3 mm depth. This shift could be resolved by installing the server and camera gripper precisely. Therefore, the obtained results indicated that the proposed system with acceptable errors can be used in industrial AOI tasks. The developed offline programming (OLP) platform is an intuitive and interactive system that does not require the physical presence of the server or the industrial manipulator, which allows non-robot-expert AOI software engineers to perform object inspection trajectory setup by only using scanned data at any location. Furthermore, this system can be used to perform the addition or change of the inspection trajectory by modifying the saved scanned information, changing cameras and lenses, and virtually changing the camera positions based on the cloud data at any location and time. To achieve the smart system, we have not built the real-time automatic feedback system, on the production line, based on the captured images for AOI functions, which can be used to detect deteriorating precision of the fixture mechanism of the object, loosening fixture of camera and lens, or worsening robot repeatability precision. The implementation of such cloud-based autonomous inspection system in the industry reduces human effort and time and increases flexibility and productivity.

## Figures and Tables

**Table 1:** Comparison between existing automatic optical inspection (AOI) systems and the proposed system.

Features	AOI Systems	Proposed System
Requires robot expert to operate	Yes	No
Setup complexity	High	Low
Requires physical presence of objects	Yes	No
Manageable from multi-locations	No	Yes
Inspection positions definition	Fixed	Flexible

**Table 2:** Sample of virtual and real-time images captured by the acA1920-25gc and a 4-mm focal lens.

	Virtual Image	Real Image
**Position 1**	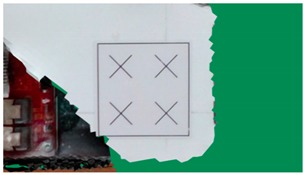	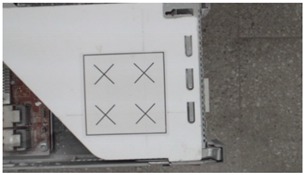
**Position 2**	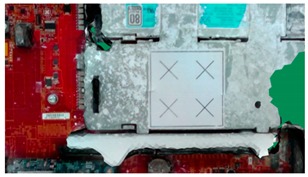	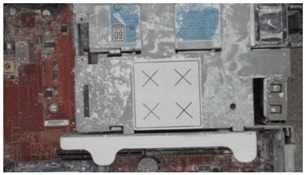
**Position 3**	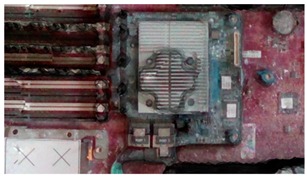	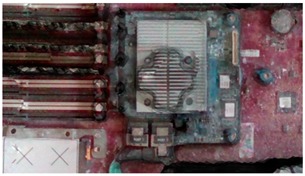
**Position 4**